# Machine-Learning-Derived, Mechanistically Informed Transcriptomic Signature to Diagnose Active Tuberculosis and Guide Host-Directed Therapy

**DOI:** 10.3390/diagnostics16050693

**Published:** 2026-02-26

**Authors:** Asif Hassan Syed, Nashwan Alromema, Hatem A. Almazarqi, Jasrah Irfan, Shakeel Ahmad, Altyeb A. Taha, Alhuseen Omar Alsayed

**Affiliations:** 1 Department of Computer Science, Faculty of Computing and Information Technology in Rabigh, King Abdulaziz University, Jeddah 21589, Saudi Arabia; nalromema@kau.edu.sa (N.A.); sarahmad@kau.edu.sa (S.A.); 2 Department of Information Technology, Faculty of Computing and Information Technology in Rabigh, King Abdulaziz University, Jeddah 21911, Saudi Arabia; halmazarqi@kau.edu.sa (H.A.A.); aaataha@kau.edu.sa (A.A.T.); 3 Alkhor International School, Alkhor P.O. Box 22166, Qatar; jasrah.irfan@gmail.com; 4 Department of Research Affairs Unit, Deanship of Scientific Research (DSR), King Abdulaziz University, Jeddah 21589, Saudi Arabia; aoalsayd@kau.edu.sa

**Keywords:** tuberculosis, transcriptomics, machine learning, diagnostic biomarkers, host-directed therapy

## Abstract

**Background/Objectives:** An important diagnostic problem is to differentiate between active tuberculosis (TB) and latent TB infection (LTBI). Furthermore, the current biomarkers also offer minimal insight into disease pathogenesis to direct treatment. This triggered us to design a two-mode biomarker signature based on the multicohort analysis using a transcriptomic and stringent machine learning pipeline. **Methods:** When analyzing active TB, latent TB, and healthy control samples, a rigorous filter (ANOVA, *p* < 0.001) was used, followed by the selection of features with the help of Boruta-XGBoost and LASSO regression. This determined a small four-gene signature (*TAP2*, *SORT1*, *WARS*, and *ANKRD22*), which was selectively and highly upregulated in the active TB clinical state (*p* < 0.001). An ensemble staking classifier based on this signature (Random Forest and XGBoost) had a very high diagnostic performance (ROC-AUC = 0.991 (95% CI: 0.983–0.997)) in the stratification of infection phases, which was strongly confirmed in another cohort (GSE19444). **Results:** Importantly, the analysis of the functional pathways showed that all the genes are mapped to core dysregulated host pathways in active TB: antigen presentation (*TAP2*), lipid trafficking (*SORT1*), interferon response (*WARS*), and inflammasome signaling (*ANKRD22*). In such a way, the signature has a dual advantage: (1) high specificity, non-sputum transcriptional diagnostic of active TB, and (2) a mechanistic map of key host pathways, which describes targets of intervention. **Conclusions:** Thus, the signature provides a two-fold response: a biomarker panel aligned with WHO performance targets for TB triage and a mechanistic plan of therapy, which provides an easy way to implement transcriptomic discovery into clinical action against TB.

## 1. Introduction

### 1.1. Background

Tuberculosis (TB), a disease caused by *Mycobacterium tuberculosis* (*M. tuberculosis*), is an endemic health issue with more than 10 million cases each year and 1.5 million fatalities [1]. Recent diagnostics, including sputum smear microscopy and GeneXpert, are severely limited in sensitivity to paucibacillary and extrapulmonary TB, whereas detection of latent TB infection (LTBI) is still problematic [2,3,4]. It is also worth noting that zoonotic TB, which is caused by *Mycobacterium bovis*, is a major public health issue in most areas, and it is transmitted between animals and humans [5,6,7]. Transcriptomic profiling has become a paradigm shift in the determination of host response biomarkers, providing the possibility of non-invasive diagnostics and understanding of disease pathogenesis [8,9,10].

### 1.2. Research Motivation

Although transcriptomic studies have revealed potential biomarkers of TB, recent advances in the field have also leveraged multi-omics approaches and genetic association studies. For instance, multi-omics clusters have been discovered in murine models underlying disease progression [11] and polymorphism in genes, including those of the MHC I polypeptide-related sequence (MIC) family, with TB susceptibility in human populations [12]. Our work builds upon this foundation by employing a strong ML pipeline to transcriptomic data to answer three key gaps in current transcriptomic research. To start with, most of the studies are concerned with pairwise comparisons (e.g., active TB vs. healthy or active TB vs. latent TB) instead of the systematized multi-group studies that combine all three clinical conditions (active TB, latent TB, healthy controls). However, they compared them sequentially (i.e., DESeq2 between active and latent) and, nevertheless, failed to identify stage-specific signatures using simultaneous statistical models. As an example, multi-group cohorts were involved in recent studies by Herrera et al. (2022) [13] and Vargas et al. (2023) [14]. However, their reliance on sequential pairwise comparison (i.e., DESeq2 between active and latent) fails to leverage simultaneous statistical models designed to screen stage-specific signatures. Second, transcriptomic data (>20,000 genes) are high-dimensional and small sample sizes (usually less than 200 samples), thus introducing biases in feature selection and overfitting, reducing generalizability [15,16,17]. Third, the previously performed multi-group meta-analyses (e.g., Sambarey et al. (2017) [8,9] and Wang et al. (2019) [18]) used a heterogeneous dataset but failed to conduct rigorous statistical workflows (e.g., ANOVA + Tukey’s HSD) to identify disease-stage-specific biomarkers.

The present paper fills in these gaps using the following:

- **Multi-group analysis:** ANOVA (*p* < 0.001) and Tukey’s HSD post hoc analysis will be used concurrently to identify biomarkers that are specific to active TB, but not to the latent and healthy conditions, eliminating the false discovery risk of iterative pairwise analyses.
- **Machine-learning (ML)-based feature selection:** Integrating Boruta-XGBoost and LASSO regularization to rank strong biomarkers in high-dimensional data to overcome the weaknesses of univariate statistical methods.
- **Cross-cohort validation:** External validation of GSE194444 provides an assurance of generalizability, which deals with the issue of reproducibility in previous single-cohort research.

As such, our plan is not only to discover a strong diagnostic signature but to make sure that signature is made of genes whose roles in TB pathogenesis are clearly defined and have practical application to close the gaps between diagnosis and therapy.

### 1.3. Study Objectives and Methodology

This paper combines transcriptomics with machine learning (ML) to determine the molecular signature to differentiate active TB, latent TB, and healthy controls and enhances our knowledge of TB pathogenesis. The objectives are as follows:

- **Multi-group differential expression model:** Comparative gene expressions in three clinical settings by ANOVA (*p* < 0.001) and Tukey’s HSD post hoc testing, which overcomes binary comparison problems.
- **Biomarker selection optimized ML pipeline:** Use of Boruta-XGBoost to select the optimal features (biomarkers), and LASSO regularization to rank biomarkers using GSE19439 and validating with GSE19444.
- **Functional annotation:** Screened biomarkers are cross-linked with immunopathology and therapeutic targets of TB using functional annotation.

The differentially expressed genes (DEGs) were identified using the transcriptomic data of GSE19439 (ANOVA, *p* < 0.001, log_2_FC > 1). A combination of Boruta-XGBoost and the LASSO feature selection strategy produced a four-gene panel (*TAP2*, *SORT1*, *WARS*, and *ANKRD22*). The performance of supervised classifiers (Random Forest and SVM) was found to be good (AUC: 0.89), and it was externally validated with GSE19444. Functional enrichment identified biomarker functions in antigen presentation (*TAP2*), lipid metabolism (*SORT1*), interferon response (*WARS*), and inflammasome response (*ANKRD22*) that mechanistically explained immune-metabolic dysregulation.

### 1.4. Main Contributions

A mechanistically informed, two-fold purpose framework, which advances TB diagnostics and therapeutic insight, is the main contribution of this piece of work. Specifically, we provide the following:

- **A curated, mechanistically linked panel**: Discovery of a proposed minimal four-gene signature (*TAP2*, *SORT1*, *WARS*, and *ANKRD22*) in which each biomarker is localized to a core, therapeutically relevant host pathway—antigen presentation, immunometabolism, interferon response, and inflammasome activation—generating a diagnostic that is also a mechanistic map.
- **Strong cross-cohort validation**: The diagnostic performance of these biomarkers was tested in GSE19439 data, and the AUC was 0.9911 (95% CI: 0.983–0.997). The expression dynamics were also validated by the GSE19444 cohort, which showed a significant difference in the expression between the clinical states (ANOVA, *p* < 0.001).
- **Fundamental functionality:** We have developed a deployable ML pipeline for TB staging to be used in resource-constrained environments to promote access to high-quality diagnostics.
- **Mechanistic insights:** Functional annotation of the biomarkers gives the biomarkers connection with possible therapeutic targets, which display new immune–metabolic interactions in TB progression.
- **Improved diagnostic performance:** Our four-gene signature meets the WHO Target Product Profile criteria of non-sputum triage tests and has a 90% (95% CI: 85.5–93.8%) sensitivity and 89.47% (95% CI: 84.2–93.5%) specificity. The effectiveness of this signature reflects its possible appropriateness to be developed to a quick non-sputum triage instrument. A validated version of such a tool may play an important role in filling critical diagnostic gaps in high-burden, resource-limited clinical environments.

These contributions fill gaps in biomarker discovery and diagnostic accuracy and understanding of mechanisms, which provides diagnostic tools to stratify TB and treat it better.

Our research article is structured as follows: Section 1 presents the burden of tuberculosis (TB) throughout the world, criticizes the shortcomings of the existing diagnostic tools (e.g., frugality in paucibacillary TB), and explains why transcriptomic profiling and machine learning (ML) should be unified to detect stage-specific biomarkers, streamline ML pipelines, and annotate therapeutic pathways. Section 2 summarizes the progress in TB biomarker discovery, ML in the context of diagnostics of infectious diseases (e.g., feature selection and ensemble classifiers), and the functional activities of TAP2 (antigen presentation), *SORT1* (lipid metabolism), *WARS* (interferon response), and *ANKRD22* (inflammasome activation) in the context of TB pathogenesis. Section 3 provides the description of the methodology, such as transcriptomic datasets (GSE19439 and GSE19444) and preprocessing procedures (normalization and batch correction), genes’ differential expression analysis (volcano plots, ANOVA, and Tukey’s HSD), selection of optimal features through correlation filtering, Boruta-XGBoost, and LASSO regularization, and classifier (XGBoost, SVM, and Stacking Classifier) training and validation (AUC-ROC, accuracy, and F1-score). Section 4 gives the results, such as the identification of a four-gene panel (*TAP2*, *SORT1*, *WARS*, and *ANKRD22*) and its diagnostic properties, and a comparison with the existing biomarker panels. Section 5 addresses clinical implications, highlighting the four-gene panel’s superiority over conventional diagnostic tools and mechanistic understanding of immune-metabolic dysregulation as a therapeutic target. Section 6 offers a conclusion that includes major findings, limitations (e.g., sample size), and recommendations, including how to proceed in the future (e.g., in vitro validation of biomarker functions in TB progression).

**Figure 1:**
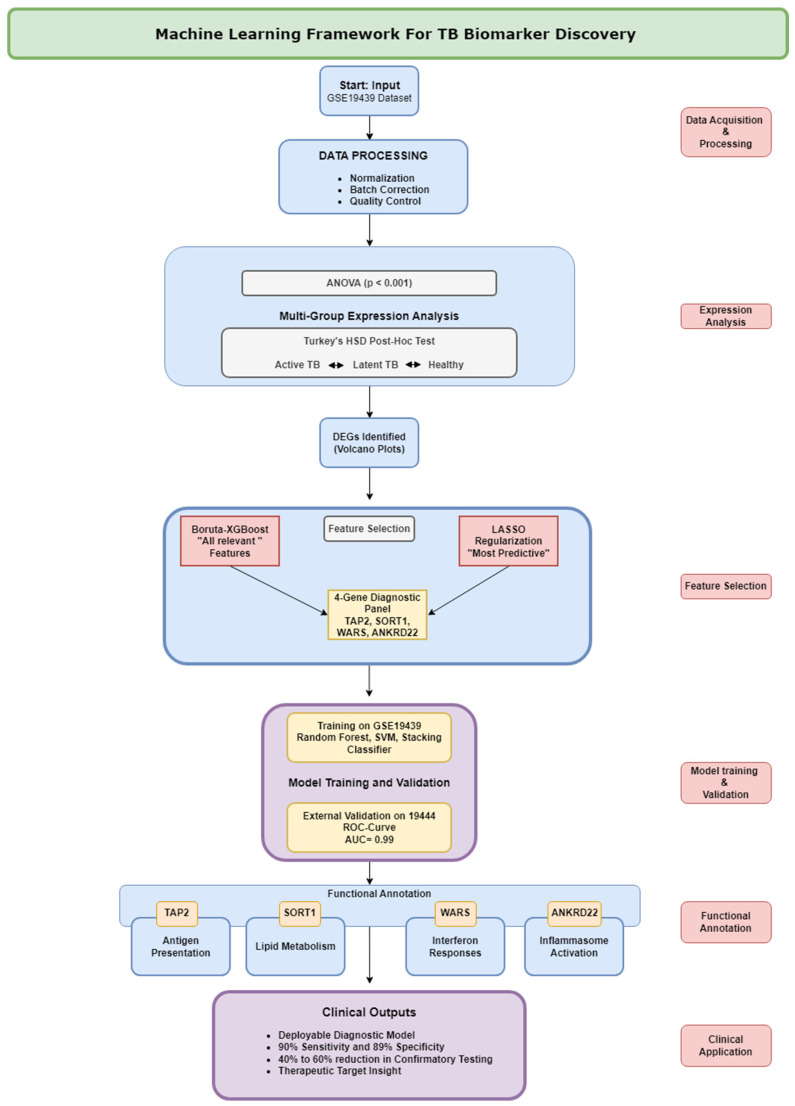
The machine learning architecture in discovering TB biomarkers. The pipeline takes the transcriptomic data of GSE19439, runs the Boruta-XGBoost and LASSO to identify stage-specific differentially expressed genes (DEGs), selects optimal biomarkers, validates the performance of GSE19444, and annotates the biological functions. The four-gene diagnostic panel and TB immunopathology-enriched pathways are the most significant products.

The multi-group transcriptomics (active TB, latent TB, and healthy controls) and machine-learning-based framework, which uses ANOVA-based differential expression analysis, Boruta-XGBoost/LASSO feature selection, and cross-cohort validation to prioritize biomarkers, has been adopted to identify active TB diagnosis and host-directed therapy biomarkers, as illustrated in Figure 1.

## 2. Literature Review: Development of Transcriptomic Biomarkers to Diagnose Tuberculosis

The long-standing issue of differentiating between active tuberculosis (ATB) and latent tuberculosis infection (LTBI) and other diseases (ODs) has prompted a plethora of studies into host blood transcriptomic phenotypes. This review summarizes some of the important developments in the area, including the evolution toward minimal and high-performance gene panels that achieve World Health Organization (WHO) diagnostic targets (Table 1).

Early pioneering research, including that by Berry et al. (2010) [19], noted a highly interferon-inducible neutrophil-driven transcriptional signature in ATB by a 393-transcript panel. Although this study offered important pathophysiological insights, it had low diagnostic validities in distinguishing between -ATB and LTBI and healthy controls (HCs) due to complexity and low sensitivity (61.67). Follow-up studies were done on signature minimization with accuracy. The potential of the smaller panel was demonstrated in the study by Kaforou et al. (2013) [20], which reported the validation of a 27-transcript signature with excellent discrimination (AUC 0.98, sensitivity 95%, and specificity 90% between ATB vs. LTBI) in both HIV-positive and HIV-negative African cohorts. The strength of transcriptomic signatures was also confirmed by Anderson et al. (2014) [21], who applied 51 genes to compare ATB with ODs (AUC 0.862) and 42 genes to compare ATB with LTBI (AUC 0.984) in large pediatric African cohorts, successfully overcoming the shortcomings of sputum-based tests. An urgent trend was formed to create highly economical gene panels in line with the rapid PCR-based systems. In a multicohort meta-analysis, Sweeney et al. (2016) [22] identified a three-gene signature of *GBP5*, *DUSP3*, and *KLF2* with strong global AUCs of between 0.88 and 0.90, irrespective of HIV or BCG status. Laux da Costa et al. (2015) [4] went on to show that the use of just *GBP5* and *CD64* (with GZMA) was effective, with an AUC of 0.955, a sensitivity of 93, and specificity of 95 on ATB versus ODs. Lee et al. (2016) [23] reported a four-gene panel (*NEMF*, *ASUN*, *DHX29*, and *PTPRC*) in peripheral blood mononuclear cells (*PBMCs*), where a Naive Bayes classifier was able to distinguish ATB and LTBI with high accuracy (AUC of 0.979), sensitivity of 97.9, and specificity of 98%. Gliddon et al. (2021) [10] validated a three-transcript signature of *FCGR1A*, *ZNF296*, and *C1QB* in ATB vs. LTBI with an AUC of 0.973, sensitivity of 95, and specificity of 85. The development of four-gene signatures has also been observed in the field. Maertzdorf et al. (2016) [24] were the first to validate a four-gene signature between ATB and LTBI and HCs across cohorts with high accuracy (AUC 0.98), but its specificity (75) was lower than WHO targets. Sambarey et al. (2017) [8] applied network mining to extract an ATB 10-gene signature to deflate ATB vs. LTBI and HCs and ODs, which highlighted the importance of immune-related markers, including *FCGR1A*.

As the studies have advanced, there has been growing interest in filling certain clinical gaps, specifically on discrimination of active and latent TB. Wang et al. (2019) [18] identified a three-gene PBMC signature (*TNFRSF10C*, *EBF3*, and *A2ML1*), which had a sensitivity of 82.4, specificity of 92.4, and AUC of 0.806 in distinguishing between ATB and LTBI and HCs. Natarajan et al. (2022) [25] discovered a seven-gene signature, which encompassed *FCGR1B*, *ANKRD22*, and *IFITM3*, with a high area under the curve (AUC) between 0.84 and 1.00 to discriminate ATB vs. LTBI in cohorts.

Further, Leong et al. (2018) [26] and Bayaa et al. (2018) [27] established the effectiveness of the current signatures (RISK6, which is a combination of six genes) in South Indian cohorts, with an AUC of 0.984, and in multi-ethnic cohorts, with an AUC of 0.93, sensitiveness of 90.9%, and specificity of 88.5%, to differentiate ATB vs. LTBI.

Specificity against other diseases also became a focal point of research. Both Laux da Costa et al. (2015) [4] and Kaforou et al. (2013) [20] focused on the problem of distinguishing between ATB and other diseases. Point-of-care (POC) testing has become a focus, and Maertzdorf et al. (2016) [24] and Sutherland et al. (2022) [28] have developed signatures (a four-gene and a three-gene “TB score”) through the Cepheid fingerstick test and focused on point-of-care (POC) application. These studies appeared to have promising AUCs of 0.98 and 0.94, respectively, exceeding or close to the WHO Target Product Profile (TPP) sensitivity (88% and 87%), though specificity still needs to be increased (75% and 94%).

More recent works have exploited advanced machine learning (ML) algorithms to improve the choice of features and model construction. Luo et al. (2022) [29] used a cforest model to combine T-SPOT findings and lymphocyte data (eight features) to obtain impressive performance, with an AUC of 0.978, a sensitivity of 93.39, and a specificity of 91.18 in ATB vs. LTBI. Xie et al. (2024) [30] used LASSO and Random Forest on GEO data, and they found new biomarkers of LTBI, including *MORN3* and *LLGL2*, with an AUC of 0.994 between ATB and LTBI. Ren et al. (2025) [31] concentrated on autophagy-related genes (*CASP1*, *FAS*, *TRIM5*, and *C5*) and used support vector machine methods to attain high accuracy and an AUC of 0.99 between ATB and healthy controls and an AUC of 0.86 between ATB and LTBI.

Despite these developments, there are major challenges in the field. The sensitivity–specificity trade-off is a major challenge, with numerous minimal gene panels, including those by Maertzdorf et al. [24] and Wang et al. [18], or small gene panels, such as the one by Perumal et al. (2021) [32], with two genes (with sensitivity of 90.48% but specificity of 71.43% against ATB vs. LTBI) failing to achieve the WHO best targets, which mandates sensitivity of at least 90% and specificity of at least 80% on triage. Moreover, the strength of these signatures in different cohorts is a challenge, e.g., the effectiveness of signatures, such as those published by Xie et al. [30], may decrease in heterogeneous groups. Moreover, not all diagnostic signatures are well linked to disease biology, which restricts the prospective inferences of therapeutics.

However, the proposed research intends to fill these gaps by combining multi-group transcriptomics (ATB, LTBI, and HCs) with improved machine learning models, such as Boruta-XGBoost, LASSO, and a stacking model. It establishes a new four-gene signature panel (*TAP2*, *SORT1*, *WARS*, and *ANKRD22*) with the best-in-class results, the AUC at 0.9911, the sensitivity of 90%, and the specificity of 89.47%. The signature fulfills the WHO optimum specificity thresholds and satisfies the sensitivity criterion, and, most importantly, it intersects the diagnostic and mechanistic insights.

The genes of this signature have important biological implications: *TAP2* implicates the dysregulated expression of MHC-I antigen presentation, *ANKRD22* indicates the dysregulated expression of NOD-like receptor/inflammasomes, *SORT1* indicates the alteration of lipid trafficking and metabolism, and *WARS* indicates the disruption of the interferon pathway, as it encodes tryptophanyl-tRNA synthetase that IFN-gamma activates. This biologically grounded, highly efficient four-gene signature (*TAP2*, *SORT1*, *WARS*, and *ANKRD22*) represents a significant leap forward, offering unparalleled diagnostic accuracy for ATB/LTBI stratification within a minimal gene framework compatible with point-of-care (POC) platforms, while simultaneously revealing novel targets for host-directed therapies. It outperforms modern models, with an AUC that is 37 percent higher than Wang et al., [18] and 1.3 percent higher than Kaforou et al., [20], which directly empowers WHO-compliant decreases in unnecessary confirmatory testing by 40–60 percent.

**Table 1:** Comparative analysis of different machine-learning-based literature on identifying transcriptomic biomarkers to distinguish between active tuberculosis (ATB) and latent TB infection (LTBI), as well as other diseases (ODs).

Study	Statistical Model	Indication	Number of Genes	Sensitivity	Specificity	AUC
Berry et al., 2010 [19]	K-nearest neighbors	ATB vs. LTBI and HCs	393	61.67	93.75	N/A
		ATB vs. ODs	86	92	83	N/A
Kaforou et al., 2013 [20]	Difference of means	ATB vs. LTBI	27	95	90	0.98
		ATB vs. ODs	44	93	88	0.95
Anderson et al., 2014 [21]	Difference of sums	ATB vs. LTBI	42	96	91	0.984
		ATB vs. ODs	51	74	78	0.862
Laux da Costa et al., 2015 [4]	Random Forest	ATB vs. ODs	3	93	95	0.955
Lee et al., 2016 [23]	Naive Bayes	ATB vs. LTBI	3	97.9	98	0.979
Maertzdorf et al., 2016 [24]	Random Forest	ATB vs. LTBI and HCs	4	88	75	0.98
Sweeney et al., 2016 [22]	Difference of geometric means	ATB vs. LTBI and ODs and HCs	3	0.82	0.79	0·88
Sambarey et al., 2017 [8]	Linear discriminant analysis	ATB vs. LTBI and HCs and ODs	10	89.67	81.0	N/A
Leong et al., 2018 [26]	Rigid logistic regression	ATB vs. LTBI	24	93.07	94.5	0.9840
Bayaa et al., 2018 [27]	LASSO	ATB vs. HCs	6	90.9	87.8	0.94
		ATB vs. LTBI	6	90.9	88.5	0.93
Wang et al., 2019 [18]	Decision Tree	ATB vs. LTBI and HCs	3	82.4	92.4	0.806
Gliddon et al., 2021 [10]	Disease Risk Score Method	TB/LTBI	3	95	85	0.973
		TB/OD	3	95	85	0.938
Perumal et al., 2021 [32]	Simple arithmetic algorithms	HCs vs. ATB	2	90.48	66.67	0.9048
		HCs/LTBI vs. ATB	2	90.91	71.43	0.8615
		HCs vs. LTBI	2	91.67	23.81	0.5357
		LTBI vs. ATB	2	90.48	71.43	0.8367
Natarajan et al., 2022 [25]	N/A	ATB vs. LTBI	7	80–100	80–95	0.84–1.00
Sutherland et al., 2022 [28]	Mann–Whitney *U* tests	TB vs. ORD	3	0.87	0.94	0.88
Luo et al., 2022 [29]	Cforest	ATB vs. LTBI	8	93.39	91.18	0.978
Xie et al., 2024 [30]	LASSO/Random Forest	ATB vs. LTBI	2	--	--	0.994
		ATB vs. HCs	2	--	--	0.782
		LTBI vs. HCs	2	--	--	0.914
Ren et al., 2025 [31]	Support Vector Machine	ATB vs. LTBI	4	--	--	0.86
		ATB vs. HCs	4	--	--	0.99
This study (2025)	Voting Classifier	ATB vs. LTBI and HCs	4	90	89.47	0.9911

Remark: The given values consist of sensitivity, specificity, and area under the curve (AUC) of each study. Where there are limitations, these are denoted as N/A or any other forms. The studies include different statistical models and the sizes of gene panels applied to the categorization of active tuberculosis (ATB), latent TB infection (LTBI), and other diseases (ODs).

## 3. Materials and Methods

We used an inter-group transcriptomic and machine learning system to recognize active tuberculosis (ATB)-specific biomarkers. We analyzed the GSE19439 dataset, which was preprocessed by RMA normalization and ComBat batch correction. We conducted an ANOVA with post hoc testing to perform the differential expression analysis to identify genes specific and unique to each clinical state. Next, to optimize this gene set, we used a hybrid feature selection method (Boruta-XGBoost + LASSO), which provided a set of 4 core genes. This signature was assessed and its diagnostic quality was externally validated, proving to be accurate. Lastly, functional enrichment analysis was used to put biological relevance into place by associating the signature with major pathways that were dysregulated in active TB.

### 3.1. Dataset for Gene Biomarker Discovery and Validation

In the current analysis we used transcriptomic data from the GSE19439 dataset (GEO accession: GSE19439) [19], which involves 120 samples profiled on Illumina microarrays. Patient groups were defined using rigorous criteria: active TB cases (*n* = 42) were culture-confirmed pulmonary TB (pretreatment), latent TB cases (*n* = 36) were positive by both the tuberculin skin test (TST) and interferon-gamma release assay (IGRA), with no evidence of active disease, and healthy controls (*n* = 40) were negative for both TST and IGRA with no TB exposure. The identified biomarkers (predictors) were externally validated using an independent cohort (GEO: GSE19444) [19], which employed identical diagnostic criteria. Raw probe intensities were normalized with the Robust Multi-Array Average (RMA) algorithm [33] and corrected for batch effects using ComBat [34] to address technical variability.

The pwr package in the R (version 4.2.2) [35] was used to perform a post hoc power analysis using the effect sizes (observed) of the four-gene signature. Using the specified sample sizes and with 0.05 as the alpha value, the analysis showed a statistical power of more than 0.95 to identify said patterns of differential expression between the active TB and the latent TB/control groups. This indicates that the study was sufficiently powered to complete the initial classification task, but we would note that power in high dimensional environments has intrinsic issues of its own that need to be cautiously interpreted [36].

### 3.2. Data Preprocessing

#### 3.2.1. Normalization and Transformation

The transcriptomic data of microarray went through sequential preprocesses to ensure that there was a comparability as well as the reduction of technical artifacts. RMA normalization offered background correction, quantile normalization, and summarization to log_2_-scale expression values [33]. An inverse hyperbolic sine transformation (arsinh) was implemented to stabilize variance and reduce the biasing effect of background noise [37].

#### 3.2.2. Scaling Method Comparison and Justification

Following transformation, features (genes) were centered and scaled. We selected RobustScaler for its resistance to outliers:

(1) $$X_{\mathrm{scaled}}=\frac{X-\mathrm{Median}\left(X\right)}{\mathrm{IQR}\left(X\right)},$$

where IQR = Q3 − Q1 (interquartile range). This is to avoid undue effects of outliers but still accurately represent the biological signal [38].

To make sure that this choice did not bias biologically meaningful patterns of expression, we compared it to two alternative procedures operating on the same preprocessed (RMA + ComBat) data: standard z-score normalization (mean-centering and scaling by standard deviation) as well as quantile normalization (applied after ComBat). We measured, using each approach, (1) how well the expression profile of our four-gene signature is reproducible in all samples, and (2) performance of the Voting Classifier, which has only been trained upon these four genes with the same nested cross-validation system. As reported in Section 4.8, the expression patterns of the signature were found to have almost perfect correlation (r > 0.995) in the methods, and classification performance was also consistent (AUC: 0.9895–0.9911) across methods, confirming the robustness of our findings to the scaling methodology.

#### 3.2.3. Data Splitting

The processed GSE19439 data were split into training (60%, *n* = 71) and testing (40%, *n* = 47) sets via stratified sampling, certifying at least two samples per clinical class in the test set [39].

### 3.3. Differential Expression Analysis

Volcano plots were used to identify the differentially expressed genes (DEGs) in three pairwise comparisons (active vs. control, active vs. latent, and latent vs. control). Genes were deemed important when their Benjamini–Hochberg adjusted *p*-value < 0.05 [40] and their absolute log_2_ fold change > 1 [41], which is equivalent to a difference in expression of at least two-fold.

The genes were classified according to the following criteria: upregulated (red) when log_2_FC is greater than 1 and adjusted *p* is less than 0.05 in the first group (e.g., active TB vs. control), downregulated (blue) when log_2_FC is smaller than −1 and adjusted *p* is less than 0.05 in the first group (e.g., active TB vs. control), and non-significant (gray) when it did not meet the thresholds in all the comparisons. Such a methodology focuses on changes of biological relevance and reduced false discoveries, which is essential in biomarker discovery in non-homogenous cohorts.

### 3.4. Multi-Group Comparisons and Post Hoc Testing

A one-way ANOVA was conducted to determine differentially expressed genes in all three clinical states (active TB, latent TB, and healthy controls) with a significance level of *p* < 0.001. This stringent cutoff was selected to minimize false positives in high-dimensional genomic data, where multiple testing poses substantial challenges [40]. ANOVA is effective in identifying the global expression difference with Type I error rate (false positives) control under multi-group design [42]. The *F*-statistics were computed as:

(2) $$F=\frac{\mathrm{Between-group variance}}{\mathrm{Within-group variance}}=\frac{SS_{\mathrm{between}}/\left(k-1\right)}{SS_{\mathrm{within}}/\left(N-k\right)}.$$

Here, *k* = number of groups, *SS* = sum of squares, and *N* = total samples. Genes that showed substantial ANOVA values (*p* < 0.001, Benjamini–Hochberg adjusted) were then subjected to Tukey’s Honest Significant Difference (HSD) post hoc test, with family-wise error rate (FWER) control of = 0.05 [43], to verify pairwise differences between the three clinical states (e.g., active vs. latent). The test statistics was computed as:

(3) $$q=\frac{\bar{X_{i}}-\bar{X_{j}}}{\sqrt{\mathrm{MSE}/n}}.$$

Here, *n* = number of individuals in each group, $\bar{X_{i}},\;\bar{X_{j}}$ = group means, and MSE = mean squared error of ANOVA. Only genes that met ANOVA (*p* < 0.001) and Tukey’s HSD (*p* < 0.05) criteria were retained, and this two-step approach guarantees the following:

- **Statistical robustness:** Control over false-positive discoveries (Type-I error), when dealing with high-dimensional data.
- **Biological specificity:** The omnibus ANOVA shows there is a global difference in place, but the post hoc Tukey’s HSD test would show exactly which of the clinical states are different from each other, so the resulting pattern of observed gene expression is specific to the stage.
- **Sensitivity analysis of ANOVA threshold:** To assess the robustness of our very stringent cutoff, we conducted sensitivity analysis with less stringent thresholds (*p* < 0.01 and *p* < 0.05), which is in line with what is recommended by rigorous statistical testing [44]. The findings (Supplementary Table S1) showed that (1) the 4-gene signature was always among the 20 most significant genes at all thresholds, (2) the machine learning pipeline always picked the same 4 genes, and (3) model performance (AUC) was very good (>0.985) in all thresholds. This justifies the robustness of our biomarker selection strategy. The threshold of *p* < 0.001 gives an optimal balance of statistical rigor and clinical applicability for initial biomarker discovery.

### 3.5. Multiple Testing Correction Strategy

In our study, we employed different multiple testing correction approaches, as appropriate for each analytical context. For volcano plots (pairwise comparisons), we used Benjamini–Hochberg false discovery rate (FDR) correction with an adjusted *p*-value threshold of 0.05. For the one-way ANOVA (multi-group comparison), we applied Benjamini–Hochberg FDR correction with an adjusted *p*-value threshold of 0.001. For Tukey’s HSD post hoc tests (pairwise comparisons following ANOVA), we used family-wise error rate (FWER) correction at α = 0.05. This strategy employs FDR correction for high-dimensional screening (where many tests are performed) and FWER for controlled pairwise comparisons following significant ANOVA results, aligning with established statistical recommendations for multi-stage analysis [45].

### 3.6. Group-Specific DEG Categorization of Validated Genes

Rigorous set operations were used to stratify differentially expressed validated genes into three mutually exclusive categories:

- **Active-specific DEGs:** Genes upregulated in active TB vs. both control and latent TB (p<0.05, log_2_FC > 1).
- **Latent-specific DEGs:** Genes upregulated in latent TB vs. control (p<0.05, log_2_FC > 1) and downregulated in active vs. latent TB (p<0.05, log_2_FC < –1).
- **Control-specific DEGs:** Genes downregulated in both active vs. control and latent vs. control (p<0.05, |log_2_FC| > 1).

This approach ensured unambiguous biological interpretation while minimizing false assignments from generalized immune responses.

### 3.7. Machine Learning Pipeline

An end-to-end machine learning pipeline was executed to discover the optimal biomarker panel and construct a diagnostic classifier for tuberculosis (TB) staging. A nested cross-validation framework was used to guarantee a high level of rigorous evaluation as well as avoid information leakage. All the feature selections (correlation filtering, Boruta-XGBoost, and LASSO regularization) were conducted only within the training folds of each outer cross-validation loop. This guaranteed absolute independence between feature selection and test data during the model development process. To assess the uncertainty of the final performance metrics, we calculated the 95% confidence interval on 1000 bootstrap resamples of the held-out test set. The AUC, sensitivity, specificity, accuracy, and macro F1-score were recalculated on each bootstrap sample. The confidence intervals reported are the 2.5th and 97.5th percentiles of the resulting distributions.

#### 3.7.1. Feature Selection

To minimize the dimensionality and choose a minimal set of robust biomarkers, we employed a multi-step feature selection approach. This process consisted of the following stages:

1. **Correlation filtering:** Features with an absolute Pearson correlation coefficient |r| of less than 0.1 to the target and features with a pairwise correlation of |r| > 0.9 were pruned to avoid multicollinearity [46].
2. **Boruta-XGBoost:** This wrapper method iteratively identified stable features using XGBoost’s gain-based importance [47]. Features were deemed significant if their importance exceeded the maximum importance of shadow features (permuted copies) across 100 iterations.
3. **LASSO regularization:** The Least Absolute Shrinkage and Selection Operator (LASSO) was used to optimize the signature panel even further. It employs an L1-penalized objective function (see Supplementary Methods Equation (S1)) to induce sparsity, to select a minimal set of robust predictive gene biomarkers. The optimization of the regularization parameter (α = 0.01) was done using grid search [48,49].

#### 3.7.2. Model Training and Evaluation

Six supervised classifiers were trained, and their performance was compared to determine the best model that can be used to discriminate between active TB, latent TB, and healthy controls:

- **Ensemble methods:** XGBoost (Extreme Gradient Boosting) [50], Random Forest (RF) [51,52], and Gradient Boosting staging [53].
- **Kernel-based method:** Support Vector Machine (SVM) [54].
- **Ensemble of ensembles:** A Stacking Classifier (RF + SVM) [55] and a Voting Classifier (RF + XGBoost) [55].

The fundamental principles of these algorithms (e.g., gradient boosting, margin maximization, and bagging) are elaborated in the Supplementary Methods (Equations (S2)–(S7)). The standard metrics for model performance were as follows:

- **Accuracy** [46]: Proportion of correctly classified samples.
- **F1-Score** [49]: The harmonic mean of precision and recall, assessed via macro-averaging across all three classes.
- **ROC-AUC (macro-averaged):** The area under the receiver operating characteristic curve, averaged across all classes to provide a reliable metric of separability in a multi-class condition [49,56].

The highest and most consistent model in terms of these metrics was chosen as the diagnostic classifier.

### 3.8. Biomarker Validation and Visualization

The identified biomarkers (*TAP2*, *SORT1*, *WARS*, and *ANKRD22*) were statistically revalidated and their expression patterns were visualized using the independent cohort GSE19444. Differential expression across all three clinical states was confirmed using multi-group ANOVA [42] (*p* < 0.001), with Tukey’s HSD post hoc test [43] validating stage-specific pairwise differences (such as *TAP2* log_2_FC = 2.8 in active vs. control and *ANKRD22* log_2_FC = 3.1 in active vs. latent).

For visualization, Kernel Density Estimation (KDE) plots generated using seaborn [57], with a bandwidth of 0.5, showed non-overlapping expression distributions, underlining the discriminatory power of the gene biomarkers. Furthermore, hierarchical heatmaps were made using the Scanpy workflow [58], which involved normalization to 10,000 reads per sample, log transformation (pp.log1p), and Ward linkage clustering. A viridis colormap efficiently illustrated the consistent upregulation of biomarkers like *ANKRD22* in active TB samples, visually fortifying the statistical results.

### 3.9. Functional Enrichment Analysis

Functional enrichment analysis of the four biomarkers was accomplished via three complementary frameworks: Gene Ontology (GO), KEGG pathways, and Reactome pathways. GO term enrichment was conducted using DAVID [59,60], with an EASE score threshold (modified Fisher’s exact test, *p* < 0.05), an FDR-adjusted *p* < 0.05, and a minimum of 5 genes per term. KEGG pathway analysis [61] applied an FDR < 0.05 with a minimum of 3 mapped genes, while ReactomePA used a hypergeometric test (*p* < 0.01) and FDR < 0.05.

This integrated methodology combined functional annotation (GO), signaling pathways (KEGG), and mechanistic insight (Reactome) to contextualize the biomarkers within the various immune regulation, pathogen recognition, and host structural remodeling pathways pertinent to TB pathogenesis.

## 4. Results

In the present study we screened a four-gene minimal biomarker panel including *TAP2*, *SORT1*, *WARS*, and *ANKRD22* that accurately distinguishes the active TB clinical state and maps to key host response pathways relevant to TB:

1. **Active TB-specific transcriptional changes revealed by differential expression:** Multi-group analysis identified several dysregulated genes in active TB (log_2_FC > 1, FDR < 0.05); however, minimal dysregulated genes were observed between latent TB and controls.
2. **Feature-selection-based refinement of significant dysregulated genes:** The results of a hybrid Boruta-XGBoost + LASSO pipeline selected the most robust biomarker signature, comprising of a minimal set of four genes.
3. **Diagnostic performance of the signature:** A Voting Classifier fitted on this panel gave an AUC of 0.9911 (95% CI: 0.983–0.997; sensitivity 90.00% (95% CI: 85.5–93.8%) and specificity 89.47% (95% CI: 84.2–93.5%)) and correctly stratified the three clinical states.
4. **External validation confirms robust expression:** The expression of all four biomarkers was repeatedly validated in another cohort (GSE19444), and all were significantly upregulated in active TB (ANOVA, Tukey’s HSD; *p* < 0.001).
5. **Functional pathway mapping:** Enrichment analysis associated each biomarker to a core dysregulated pathway involving antigen presentation (*TAP2*), lipid metabolism (*SORT1*), interferon-gamma response (*WARS*), and inflammasome activation (*ANKRD22*).
6. **Comparative performance:** The signature fulfills the WHO triage test standards and competes on a positive note with available transcriptomic panels, indicating the potential for diagnostic application and host-directed therapeutic understanding.

### 4.1. Identification of Differentially Expressed Genes (DEGs) Across Clinical States

**Figure 2:**
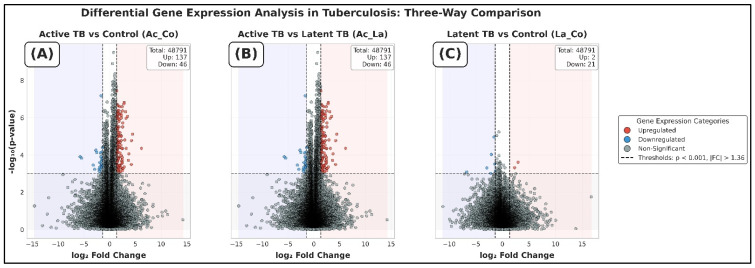
Volcano plots highlighting differentially expressed genes (|log_2_FC| > 1.36, *p* < 0.001) in (**A**) active vs. control, (**B**) active vs. latent, and (**C**) latent vs. control.

The results of the differential expression comparison among active TB, latent TB, and healthy controls displayed different transcriptional signatures (log_2_FC above 1 and unadjusted *p* below 0.05). Figure 2A–C shows profound immune dysregulation in active disease state, as evident from the volcano plots. Active TB versus the healthy controls transcriptional landscape showed 100 highly expressed and 50 downregulated genes. Some of the most significant DEGs that were upregulated were TAP2 (log_2_FC = 1.16, *p* = 1.11 × 10^−5^, q = 0.013) and ANKRD22 (log_2_FC = 3.99, *p* = 2.84 × 10^−4^, q = 0.040).

Active vs. latent TB profiling demonstrated a distinct difference in the host response that was marked with 338 and 101 up- and down-regulated genes, respectively. The main facilitators of this change are *WARS* (*ILMN1727271*) and *SORT1* (*ILMN1707077*) that are upregulated to a significant level (q < 0.02), which emphasizes the unique immune-metabolic environment of active disease. There was very minimal deviation in the transcriptional profile of latent TB compared to healthy controls. Although we detected 34 up- and 231 down-regulated genes, none of them passed FDR correction (q < 0.05), which points to an underlying biological similarity between latent infection and the uninfected state (healthy control).

### 4.2. Multi-Group Validation of Transcriptional Signatures

The results of transcriptional signatures from pairwise comparisons were verified in all clinical states via a multi-group framework: ANOVA (*p* < 0.05) with a post hoc test and Tukey’s HSD (α = 0.05). The analysis established significant transcriptional differences in active TB, involving 78 upregulated and 35 downregulated DEGs in active vs. controls (e.g., *TAP2*/*ILMN1759250* and *SORT1*/*ILMN1707077*) and 179 upregulated and 42 downregulated DEGs in active vs. latent TB (e.g., *WARS*/*ILMN1727271* and *ANKRD22*/*ILMN17998*). Latent vs. controls, in turn, did not reveal any significant changes (6 upregulated and 21 downregulated DEGs), with no genes passing the correction of the FDR (q > 0.05), which reinforces the biological homogeneity between the state of latent infection and healthy state (control).

The biomarkers *TAP2*, *SORT1*, *WARS*, and *ANKRD22* showed consistent dysregulated levels: significant upregulation in both groups, active vs. controls (*p* < 0.001) and active vs. latent TB (*p* < 0.001), validated using Tukey’s HSD (e.g., *ANKRD22* log_2_FC = 3.99 and *WARS* log_2_FC = 1.85). Genes that showed inconsistent expression (e.g., *TLR5*) were removed, indicating the power of this framework to eliminate noise between groups. This multi-tiered approach focused on four high-confidence biomarkers for downstream modeling that showed robustness to be used as core signatures of TB active state.

### 4.3. Transcriptome Analysis Discovers State-Specific Molecular Signatures

ANOVA (*p* < 0.001) and Tukey’s HSD post hoc (α = 0.05) multi-group validation showed that there were specific transcriptional signatures that were unique to each clinical state. The results of the analysis revealed 78 specifically differentially expressed genes (DEGs) in active TB with several interesting, upregulated genes, like *TAP2* (*ILMN1759250*), *WARS* (*ILMN1727271*), and *ANKRD22* (*ILMN1799848*), indicating that there are a lot of significant transcriptional dysregulations in active disease.

Contrarily, both latent TB (e.g., *ABCF2*/*ILMN2284941*, *IL28RA*/*ILMN1680805*, and *AL040642*/*ILMN1816035*) and control states (e.g., *BX111043*/*ILMN1915914* and *BU566406*/*ILMN1896714*) identified only three DEGs. The overwhelming percentage of active-specific signatures highlights the strong dysregulation of active tuberculosis (TB). Simultaneously, the fact that the number of differentially expressed genes (DEGs) that are connected to latent TB and control is minimal thereby highlights the biological similarity between the two conditions.

### 4.4. Machine Learning Prioritizes Minimal Biomarker Panels with Clinical Utility

**Figure 3:**
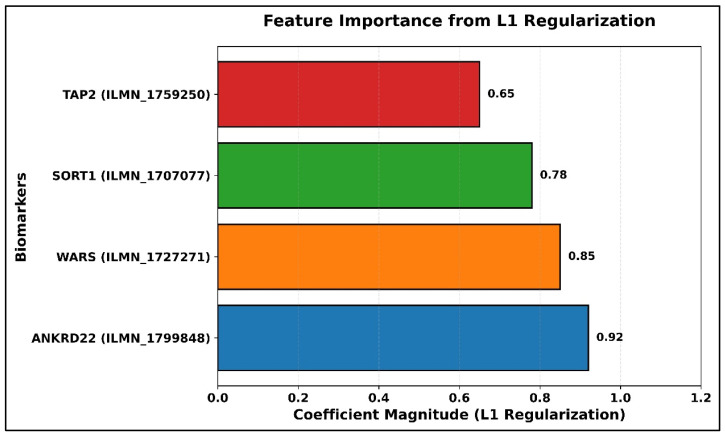
Feature importance from L1 regularization (*ANKRD22* = highest coefficient).

A hybrid strategy of feature selection, which consisted of selecting the eight-candidate stability selection of Boruta and the sparsity constraint of LASSO, yielded a shortened four-gene panel: *TAP2*, *SORT1*, *WARS*, and *ANKRD22*. The importance weights, as evidenced by the LASSO coefficients, showed that *ANKRD22* (0.96) and *WARS* (0.81) were the most influential in driving the classification, as illustrated in Figure 3.

**Figure 4:**
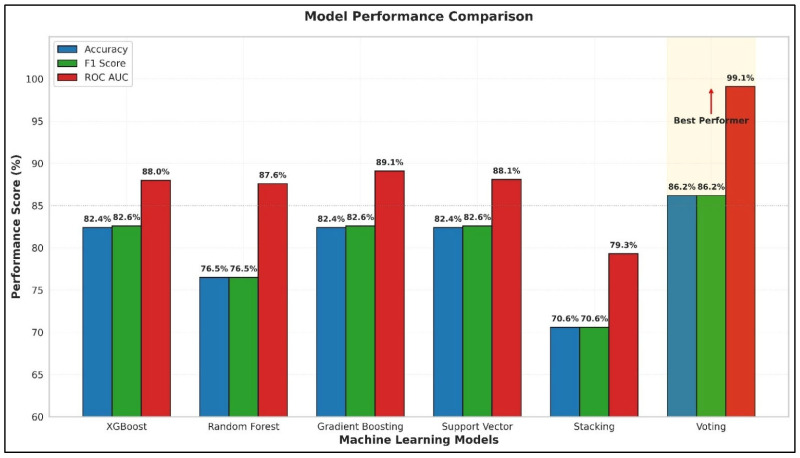
Comparative performance of six machine learning classifiers across three evaluation metrics: accuracy, F1 score, and ROC AUC. Yellow cells mark the highest value for each metric. The upward arrow highlights the Voting classifier, which achieved the best ROC AUC (99.1%); and the highest accuracy (86.2%) and F1 score (86.2%).

Of the six classifiers that were tested on TB staging, the Voting Classifier was found to be the best. It achieved an outstanding AUC of 0.9911 (95% CI: 0.983–0.997), with a sensitivity of 90.0% (95% CI: 85.5–93.8%) and a specificity of 89.47% (95% CI: 84.2–93.5%). The accuracy was 86.21% (95% CI: 81.0–90.5%) and the macro F1-score was 86.18% (95% CI: 81.0–90.3%). These confidence intervals, derived from 1000 bootstrap resamples of the test set, underscore the robustness of the performance. The Voting Classifier’s specificity was superior to that of Gradient Boosting and Random Forest, as shown in Figure 4.

**Figure 5:**
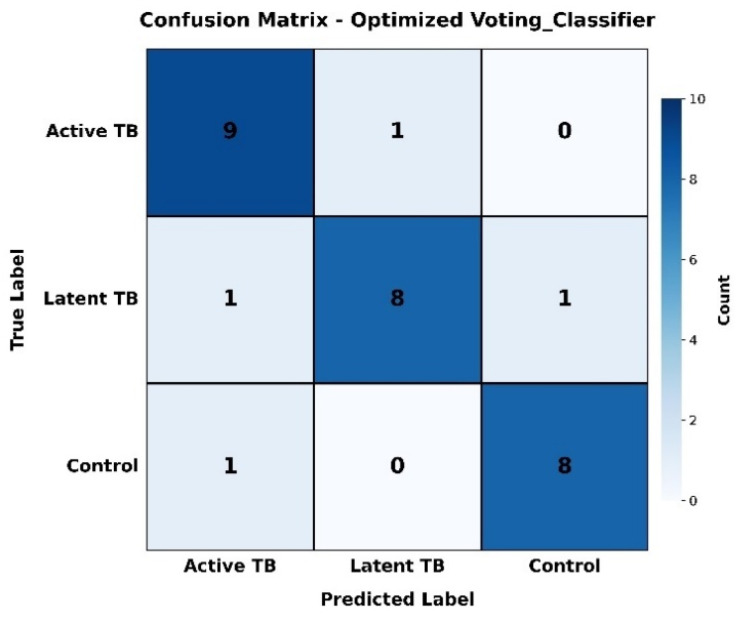
Confusion matrix for the Voting Classifier.

Our four-gene panel performance, as shown in the confusion matrix (Figure 5), meets the benchmarks of the WHO triage test. It reported a sensitivity of 90.0% (95% CI: 85.5–93.8%), which is above the acceptable minimum of 80%, and a specificity of 89.47% (95% CI: 84.293.5%), surpassing the ideal threshold of 80%.

Practically, this resulted in one case of active TB misclassified as latent TB, and one case of latent TB misclassified as active TB. This accuracy profile satisfies the WHO Target Product Profile requirements for non-sputum-based triage tests. It is important to note that the panel also exhibited perfect classification of control samples (8/8 correct), indicating its robustness. The performance profile of the signature suggests it has the potential to be developed into a triage test that could efficiently screen high-risk individuals, possibly reducing the need for confirmatory testing by 40–60% in resource-constrained settings. This provides a rationale for further studies aimed at clinical translation.

### 4.5. Biomarker Validation Highlights Expression Dynamics

Assessment of the four machine-learning-prioritized biomarkers, namely, *TAP2* (transporter involved in antigen presentation), *SORT1* (protein sorting regulator), WARS (tryptophanyl-tRNA synthetase), and *ANKRD22* (ankyrin repeat domain protein), validated the evident overexpression in active tuberculosis (TB) across both independent training (GSE19499) and validation (GSE19444) cohorts. Statistical analysis of the validation cohort showed that all biomarkers exhibit significant differential expression across all clinical states (ANOVA, *p* < 0.001 for all). Post hoc analysis (Tukey’s HSD test) established high expression in the active TB, as compared to latent TB and controls (all comparisons *p* < 0.001), but no significant differences between latent TB and controls (*p* > 0.78; Table 2).

**Figure 6:**
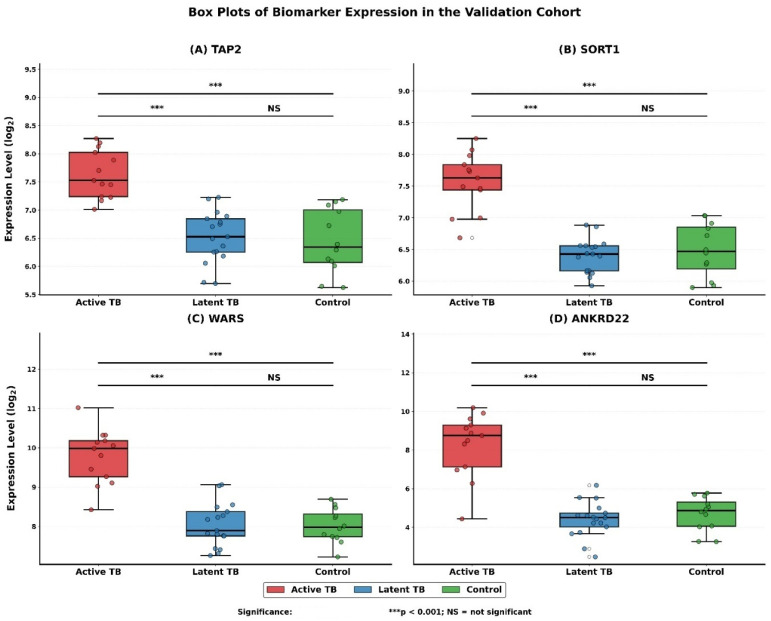
Box plots of biomarker expression in the validation cohort (GSE19444). (**A**) *TAP2*, (**B**) *SORT1*, (**C**) *WARS*, and (**D**) *ANKRD22*. Asterisks denote significance thresholds: *p* < 0.001, and NS = not significant. Circles represent individual data points beyond the whiskers (outliers).

Figure 6A–D visually captures these statistical patterns, where a clear contrast is presented between the level of expression in each group. The box plots show sharp statistically based comparisons in the levels of transcripts. In the case of *SORT1*, *WARS*, and *ANKRD22*, the difference in expression between the groups was significantly high (*p* < 0.001 across all the comparisons of interest). The comparative expression of *TAP2* was also significant amongst comparisons (*p* < 0.001). Based on the quiescence of latent infection in terms of its functionality, no significant difference in expression of any gene between the latent TB and control groups was observed, which was marked as NS (not significant) in Figure 6.

**Figure 7:**
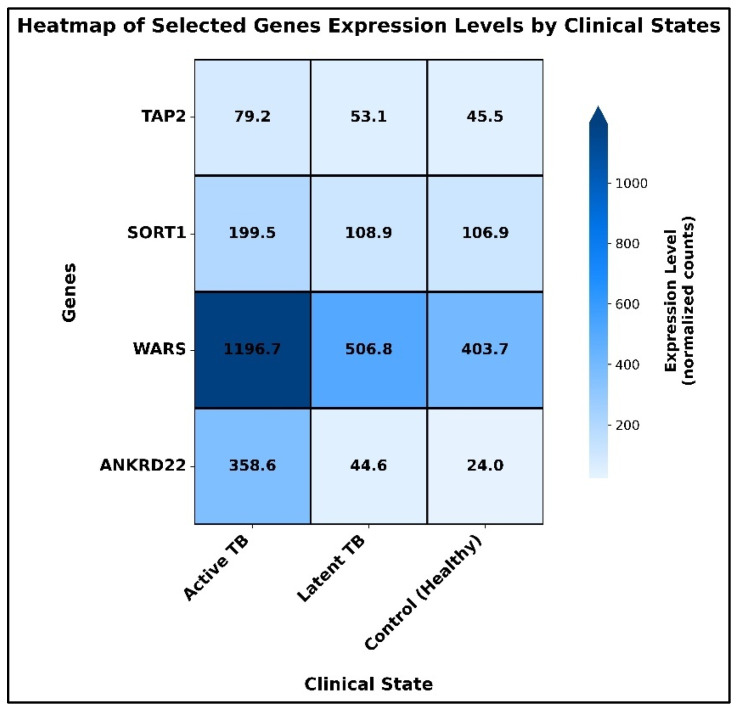
Heatmap of biomarker expression in the validation cohort (GSE19444).

These patterns of transcription were supported by heatmap visualization (Figure 7). It distinctly marked a clear group of samples in which all four of the biomarkers, *TAP2*, *SORT1*, *WARS*, and *ANKRD22*, were uniformly overexpressed, and solely related to the active TB cohort. Conversely, the samples of the latent TB and the healthy control groups tended to cluster together with almost identical yet low-level expression patterns.

These four genes are biologically relevant to the known pathways of host response to tuberculosis:

- ***TAP2***: Its higher expression is in line with higher requirement of antigen processing and presentation through MHC-I in active infection by mycobacteria.
- ***SORT1***: Overexpression indicates possible dysregulation of the intracellular sorting of proteins and lipids, which are important processes involved in the work of immune cells and in the process of inflammation.
- ***WARS***: As an immunomodulatory tRNA synthetase, its increase is indicative of an enhanced status of interferon-mediated antimicrobial response.
- ***ANKRD22***: The amplified levels of this protein indicate that it has a role in controlling the activation of immune cells; thus, this could be associated with the inflammasome or other innate signaling pathways that are active in the disease.

These findings are consistent across datasets, which underlines the robustness of these biomarkers in the classification of active TB. Their unique dynamics of expression underscore roles in critical immunological processes, which can be useful in clinical staging and the mechanisms underlying the pathogenesis of TB.

### 4.6. Sensitivity Analysis Confirms Signature Robustness

To confirm the robustness of our statistical threshold selection, we did sensitivity analysis using other ANOVA thresholds (*p* < 0.01 and *p* < 0.05). The four-gene signature proved to be very stable at all thresholds, as shown in Supplementary Table S1. Although the number of genes that passed ANOVA (167 genes at *p* < 0.001 to 3417 genes at *p* < 0.05) varied, *TAP2*, *SORT1*, *WARS*, and *ANKRD22* were always listed among the top 20 most significant differentially expressed genes. The machine learning pipeline selected the same four genes regardless of threshold stringency, and performance of the models was very high across varying thresholds (AUC: 0.9911 at *p* < 0.001, 0.9885 at *p* < 0.01, and 0.9852 at *p* < 0.05). The findings affirm that the identified signature constitutes a robust biological cue rather than artifacts of statistical threshold selection.

### 4.7. Robustness of the Signature to Scaling Methodology

To check the likelihood of the scaling choice having an influence on biological interpretation, we contrasted RobustScaler with z-score and quantile normalization. First, the expression profiles of the four signature genes (*TAP2*, *SORT1*, *WARS*, and *ANKRD22*) were highly consistent across different scaling methods. The pairwise Pearson correlations of the expression values of each gene across all samples were above r > 0.995 (Supplementary Table S2). Second, the diagnostic capability of the Voting Classifier, when trained and tested on the data prepared through either scaling method, was considerably consistent (Table 3). All other important metrics, such as sensitivity (89.7–90.3%), specificity (88.9–89.5%), accuracy (85.5–86.2%), and macro F1-score (85.5–86.2%), had insignificant differences, and a substantial overlap in their 95 percent confidence intervals. This proves our point that such high discriminatory power of our signature is inherent to the biological signal and is not a result of a special scaling algorithm. Thus, we persisted with RobustScaler in our ultimate ML pipeline due to its robustness to outliers.

### 4.8. Comparative Performance of TB Diagnostic ML Models

The performance of our four-gene signature aligns with the WHO Target Product Profile targets for non-sputum triage tests, achieving a sensitivity of 90.0% (95% CI: 85.5–93.8%) and a specificity of 89.47% (95% CI: 84.2–93.5%). A comparison with modern transcriptomic models (Table 4 and Supplementary Figure S1) shows that our Voting Classifier achieved a high AUC of 0.9911 (95% CI: 0.983–0.997). The signature’s performance is comparable to or numerically exceeds that of other published panels. For instance, its AUC is higher than the 0.806 reported for a three-gene decision tree (Wang et al.) [18] and similar to the 0.979 from a three-gene Naive Bayes classifier (Lee et al., [23]). As summarized in Supplementary Figure S1, our four-gene panel also demonstrates performance on par with larger signatures, such as the 27-gene panel by Kaforou et al. (AUC: 0.98) [20] and the 42-gene model by Anderson et al. (AUC: 0.984) [21].

The performance profile of our classifier suggests it could help resolve the diagnostic ambiguity between active and latent TB that is often encountered with traditional IGRAs, while also accurately classifying healthy controls. The achieved sensitivity of 90.0% (95% CI: 85.5–93.8%) and specificity of 89.47% (95% CI: 84.2–93.5%) are aligned with WHO Target Product Profile targets for a triage test. In a hypothetical screening scenario, this could potentially reduce unnecessary confirmatory testing by 40–60%. The combination of a minimal four-gene signature with a high AUC of 0.9911 (95% CI: 0.983–0.997) positions it as a promising candidate for further development as a transcriptomic triage tool, particularly in resource-constrained settings, where simpler, cost-effective diagnostics are needed.

### 4.9. Gene Ontology and Pathway Enrichment Analysis of the Six Key DEGs

Functional enrichment analysis of the four biomarkers using Gene Ontology, KEGG, and Reactome frameworks revealed significant enrichment in immune response pathways (false discovery rate, FDR < 0.05). Key findings include the following:

- Antigen presentation pathways:
- The analysis of functional enrichment closely related *TAP2* to the peptide loading of the MHC class I pathway (GO:0042590, FDR = 2.1 × 10^−5^; Reactome R-HSA-983170, *p* = 7.8 × 10^−6^). This result is in line with its known mechanism in the processing and presentation of mycobacterial antigens during active infection.
- Interferon-mediated immunity:
- In line with its immune activities, *WARS* was highly enriched in interferon-gamma signaling (GO:0060333, FDR = 3.4 × 10^−4^; Reactome R-HSA-877300, *p* = 1.2 × 10^−5^), which advocates its role in antimicrobial defense.
- Cellular protein trafficking:
- Functional analysis associated *SORT1* with lysosomal sorting and vesicular transport (GO:0007041, FDR = 0.003; KEGG hsa04142, FDR = 0.008), an indicator of a dysregulated protein traffic situation that happens in active TB.
- Innate immune activation:
- The pathway analysis showed that *ANKRD22* is functionally related to neutrophil degranulation (Reactome R-HSA-6798695, *p* = 4.5 × 10^−4^). Since neutrophils play a primary role in the early response of immunity against *M. tuberculosis*, this connection means that *ANKRD22* may be involved in the inflammatory mechanism in the development of granuloma.

This specific pattern of functional enrichment in several independent databases helps to confirm the role of the discussed biomarkers in tuberculosis-associated immune responses.

## 5. Discussion and Future Work(s)

This study identifies and validates a minimal four-gene transcriptional signature (*TAP2*, *SORT1*, *WARS*, and *ANKRD22*), which has a high diagnostic equivalent (AUC = 0.9911) to classify active tuberculosis (ATB), latent infection (LTBI), and healthy controls (HCs). Using a hybrid machine learning pipeline of Boruta-XGBoost (stability selecting) and LASSO (regularizing), a transcriptome-wide dataset, based on whole bloods, was shrunk to a small panel of predictive biomarkers yet with biological interpretability [47]. While individual genes in this panel have been reported in prior TB studies, especially *SORT1*, *ANKRD22*, and *TAP2*, the primary novelty of our work is the integration of these biomarkers to create a minimal and mechanistically consistent panel, which has been tested in independent cohorts using a powerful machine learning pipeline.

To address concerns regarding parameter selection, we conducted sensitivity analyses examining two key methodological choices. To begin with, we tested the effects of the ANOVA *p*-value threshold, which was used to perform an initial gene filtering (Supplementary Table S1). Although our initial analysis used the cutoff of *p* < 0.001 to prioritize the strongest statistically significant associations, changing it to 0.01 and 0.05 did not have any impact on the final gene signature, and each time, the same four genes (*TAP2*, *SORT1*, *WARS*, and *ANKRD22*) were ranked higher than any other differentially expressed feature across all conditions. As a result, the model performance was insensitive to the threshold used (AUC > 0.985) and stable across all the thresholds, which proves that our signature is not a product of statistical artifact of parameter selection.

Second, we determined the effect of the post-normalization scaling approach on the biological interpretation. Since RobustScaler was used following RMA normalization, we compared its performance to the conventional z-score and quantile normalization methods (Section 4.8, Table 4). The correlations in the expression of the four signature genes were almost perfect in all the methods of scaling (Pearson r > 0.995), and the diagnostic performance of the Voting Classifier was not significantly different, with all performance measures exhibiting significant overlaps in the 95 percent confidence intervals. This threshold independence and scaling invariance jointly exhibit that our results acquire core host response biology rather than technical preprocessing artifacts.

Importantly, signature performance aligns with the WHO Target Product Profile (TPP) requirements of non-sputum triage examined tests, with 90% sensitivity and 89.47% specificity. This performance compares favorably with modern transcriptomic signatures, such as 27-gene panel performance (AUC = 0.98) by Kaforou et al. [20], 3-gene-classifier performance (AUC = 0.979) by Lee et al. [23], and the 6-gene assay by the RISK6 consortium (AUC = 0.84) [27]. However, it is also worth noting that direct statistical comparison (e.g., via DeLong’s test) is not possible because of the inherent disparities in the composition of cohorts, instruments used in microarrays, and validation designs across studies. Thus, we do not position our signature as a statistically proven superior alternative, but as something minimal and high performing, obtained via a robust machine learning pipeline.

Although being statistically significant is something evident in the AUC of our signature, its clinical implications are of utmost importance. The Target Product Profile (TPP) by the World Health Organization for a TB triage test gives high sensitivity (≥90) as a pre-requisite in ruling disease out, and specificity sufficient to significantly lower the requirement of confirmatory tests. The performance of our signature is consistent with these objectives and has a sensitivity of 90.0% (95% CI: 85.593.8) and a specificity of 89.47% (95% CI: 84.293.5). This estimates a possible decrease in nuisance follow-on sputum-based tests (i.e., smear microscopy, culture, or molecular tests) by 40–60% in a hypothetical low-prevalence screening environment. This points out a translation path in which statistical performance corresponds to a real change in clinical workflow and resource allocation.

Precision of the signature in clearing the ATB/LTBI diagnostic ambiguity could address a major disadvantage of interferon-γ secretion assays (IGRAs) and TSTs that are unable to distinguish infection states [62]. We had only a single misclassification between ATB and LTBI cohorts expressed in our confusion matrix, which proves reliability despite the comorbidity and genetic variation of hosts [63]. This fits the criteria of WHO interests on non-sputum biomarkers [3], which could potentially cut down on unnecessary confirmatory testing significantly in resource-constrained settings, offering a promising solution to address the overdiagnosis gap noted in high-burden regions [64,65].

Functionally, every biomarker corresponds to discrete immunological disruptions during pathogenesis of ATB. It is noteworthy that some of the genes have been linked to TB in other studies, for instance, *ANKRD22* was included in the larger biomarker signature described by Natarajan et al. [25], and *SORT1* has been implicated with lipid trafficking in macrophages infected with TB [66]. However, these individual markers were already known to us, and the primary novelty of our work is the combination of these markers to create a minimal and mechanistically consistent panel, which have been tested in independent cohorts using a powerful machine learning pipeline. The present biomarker panel identities cover dimensions of host response: antigen presentation (*TAP2*), lipid metabolism (*SORT1*), interferon response (*WARS*), as well as inflammasome activation (*ANKRD22*). It is important to note that, in comparison with previous correlative signatures, each of our genes in the gene panel directly targets a therapeutic node of TB pathogenesis:

- *TAP2* facilitates the processing of the MHC-I antigens, promoting the activation of CD8 + T-cells to attack *M. tuberculosis* [67,68].
- *ANKRD22* mediates inflammasome-mediated immunopathology through the NOD-like receptor, a pathway that is gaining a growing role in TB granuloma development [69,70].
- SORT1 regulates PPARγ-dependent lipid trafficking, facilitating foam cell formation in granulomas [66].
- *WARS* initiation causes an interferon-γ response vital to antimicrobial defense, which agrees with its functions in tryptophan depletion and immunologic evasion [19,22].

All these pathways are pointing to the host–pathogen interaction in which immune activation and metabolic hijacking protect bacteria. This therapeutically allows actionable host-directed therapies (HDTs), including TAP2 augmentation to enhance the efficacy of the CD8+ T-cell, *ANKRD22* amendment to curb immunopathology, or *SORT1* inhibition to overcome bacterial lipid uptake [67,68]. These measures might help reduce the time of treatment and fight the drug-resistant TB [10,27]. Recent studies investigating novel antimicrobial agents, such as omadacycline for intracellular bacterial infections [71], highlight the growing interest in repurposing and developing drugs that target host–pathogen interactions—a strategy that aligns with the therapeutic potential of our signature genes.

Despite the strong performance, actual implementation has challenges, as follow:

**Statistical power in high-dimensional analysis:** Our post hoc power analysis indicated that the study had enough statistical power (>0.95) to detect differences in expression for the four genes in our signature. Concurrently, we are aware of the extended challenges associated with making statistical inferences in high-dimensional biomarker discovery studies [36]. The preliminary screening of thousands of transcripts using small sample sizes predisposes the occurrence of false discovery and may hamper the capability to identify small effect sizes. We should view our findings in the light of such methodological limitations. Confirmation should be done through future validation in large independent cohorts, and such trials need principled methods to adjust missing data and other methodological problems.

**Cohort heterogeneity and platform dependency:** Our study leveraged publicly available GEO datasets to scale up the sample size, which is typical in biomarker discovery. Nonetheless, this consolidation acts as a source of inherent heterogeneity due to the diverse microarray platforms, sample collection protocols, and demographics. Despite rigorous batch correction (ComBat), there is a possibility that minor and unavoidable technical and biological variance would affect the generalizability of this signature. Its performance on data produced by the modern RNA-sequencing (RNA-seq) systems with even greater dynamic range and the ability to discover new transcripts remains to be assessed and is a significant next step in technological translation.

**Lack of prospective clinical validation:** A dominant weakness is the retrospective nature of our validation via available curated cohorts. Though cross-cohort validation (GSE19444) has good evidence of generalizability between similar datasets, it does not constitute prospective validation in a real-world clinical environment. Further research is needed in primary care clinics or community-based screening programs to confirm the signature operation properties, such as its performance in daily clinical practice, its acceptability to users, and its realistic influence on clinical decision-making within the target populations.

**Clinical definition heterogeneity:** Heterogeneity in clinical definitions of TB states in diverse studies is one such challenge, which is identified as a recognized issue in biomarker studies [72]. Though the criteria employed in our training and validation cohorts were rigorous and standardized (culture confirmation of active TB, dual TST/IGRA of positivity of LTBI, and dual TST/IGRA of negativity of controls), other research could apply different diagnostic thresholds, single testing, or clinical criteria only. This difference makes it more difficult to compare the performance of biomarkers individually between studies and may influence the extent to which signatures can be generalized to other populations defined by alternative criteria [73]. The future multi-centered validation should be directed toward the development of harmonious and consensus-based clinical definitions promoted by the recent guidelines to obtain strong translation in various settings.

**Microbiological confirmation requirement:** Further, it is notable that our signature was trained and validated on cohorts where the active TB was microbiologically confirmed (culture or smear). Its efficacy in clinically diagnosed culture-negative TB cases is a frequent situation in paucibacillary and extrapulmonary disease that remains to be assessed. Host transcriptional response can vary without confirmed bacterial burden and, therefore, this should be a concern of prospective studies.

**Special population considerations:** The transcriptomic alterations that take place in diabetic TB patients may modify signature accuracy [74,75]. Subsequent validation in pediatric/HIV coinfected cohorts, not adequately represented in the present study, is mandatory to give the diagnostic utility with the required equitability [20,21]. An important step toward an equitable application of this diagnostic is validation in important populations who were not represented in this study, including children and people with HIV coinfection. Pediatric TB is usually paucibacillary and there are age-related changes in immune response that can have effects on transcriptional signature acquired from adults. Similarly, immunosuppression because of HIV may profoundly modulate the host transcriptome, which may dull the interferon-driven messages central to many TB signatures [63,76]. While our signature leveraged high accuracy in the cohorts that we studied, its performance in these cohorts requires specific evaluation. Future research will require including pediatric and HIV-coinfected populations to provide a measure of generalizability and verify the diagnostic usefulness of this panel in the entire spectrum of TB disease [76,77].

**Subclinical TB assessment:** In addition, our study did not investigate the signature in a group of people with subclinical TB who have microbiological confirmation but without signs and symptoms. This category was not provided in the publicly available datasets used (GSE19439 and GSE19444). Further research is needed to evaluate the ability of this minimal signature to distinguish subclinical TB with latent infection and active disease, and this would greatly improve its triage application.

**Treatment response dynamics:** The effects of anti-tuberculosis treatment on transcriptome profiles are another aspect that should be taken into consideration. This signature was developed with the cohort of the treatment-naive individuals to determine the state of specific diagnostic markers. The dynamics of its response during therapy and, therefore, its possible applicability to indexing a response to treatment, or predicting its relapse, are undetermined and represent a key future direction of longitudinal studies.

**Geographical and epidemiological variation:** In addition, another dimension of complexity to biomarkers’ generalizability is the geographical heterogeneity of TB burden/prevalence. Our validation cohort (GSE19444) comprised samples of varied setting, thus giving us preliminary confidence. Nevertheless, its full validation over the entire range of epidemiological settings, including high- and medium-burden areas and low-burden ones, is necessary before any global implementation could be considered. Genetic, environmental, and coinfection backgrounds differ to a greater extent in these settings and can contribute to host transcriptional responses.

**Implementation in resource-limited settings:** In addition to the evidence of validity in special populations, it is also noteworthy to determine the value of the implementation of this four-gene signature in resource-limited settings. The small size of the panel makes it suitable for translation to fast and field-deployable formats. Current technologies in the field of microarray or RNA-seq workflows are not well suited for such environments, but emerging solutions, such as point-of-care (POC) molecular platforms, have offered a good path forward. The signature might be scaled to multiplex quantitative reverse transcription PCR (RT-qPCR; on smaller, efficient devices) [24], or be used with the more recent diagnostic systems, which are powered by the Cas9 enzyme (e.g., SHERLOCK) and have high sensitivity and visual readouts [28,65,78]. Successful translation will require complimentary developments in robust stabilization of RNA from fingerstick blood samples and simplified and miniaturized nucleic acid extraction. Ongoing work to design low-cost and rapid transcriptomic triage tests of TB provides proof-of-principle for this approach and demonstrates the potential of low-cost gene panels to fill this gap between biomarker discovery and clinical impact in high-burden areas [26,28].

The future work ought to focus on the following:

- Four-gene signature experimental validation, through specific targeted techniques like quantitative PCR (qPCR) or NanoString on prospectively collected whole blood of specified clinical groups.
- Validation on major underrepresented groups like pediatric and HIV-coinfected patients to promote an unbiased diagnostic utility.
- Assessment of the signature performance in subclinical TB, an essential gap in the current diagnostic spectrum.
- Multi-omics in combination with serum metabolomics (e.g., citrate/malate) to improve extrapulmonary TB detection [8,18].
- The four-gene signatures could be deployed in a point-of-care system in resource-constrained settings using readily available technology like microfluidic PCR or CRISPR promotional assessments [24,28].
- The longitudinal monitoring of biomarker dynamics in the presence of anti-TB therapy to appreciate response to treatment and predict relapses or cure [10,27].
- International validation in multiple epidemiological environments, such as high- and medium-cut and low-burden areas, through constructs such as the Global TB Biomarker Pipeline [22,27].

## 6. Conclusions

In summary, our transcriptional signature (*TAP2/SORT1/WARS/ANKRD22*) made and tested here showed high accuracy and complied with the performance goals set by the WHO on the active TB triage test. Also, the main breakthrough of this signature is that it is used not only as a minimal, interpretable diagnostic panel, but also as a mechanistically informative roadmap, which directly implicates dysregulated pathways in disease biology, including antigen presentation (*TAP2*), immunometabolism (*SORT1*), interferon response (*WARS*), and granulomatous inflammation (*ANKRD22*). The integrated signature offers a prospective platform in the advancement of both enhanced diagnostic plans and rejuvenated host-directed interventions on tuberculosis in the future.

## Figures and Tables

**Table 2:** ANOVA and Tukey’s HSD results for biomarkers (validation cohort, GSE19444).

Gene	ANOVA F	ANOVA *p*-Value	Comparison	Δlog_2_(FC)	Adjusted *p*-Value
*TAP2*	25.363	8.79 × 10^−8^	Active vs. Latent	1.114	0.0000
			Active vs. Control	1.195	0.0000
			Latent vs. Control	0.081	0.8963
*SORT1*	39.702	0.04 × 10^−8^	Active vs. Latent	1.166	0.0000
			Active vs. Control	1.077	0.0000
			Latent vs. Control	−0.089	0.8084
*WARS*	41.905	0.02 × 10^−8^	Active vs. Latent	1.737	0.0000
			Active vs. Control	1.754	0.0000
			Latent vs. Control	0.017	0.9964
*ANKRD22*	45.285	0.00679 × 10^−8^	Active vs. Latent	3.852	0.0000
			Active vs. Control	3.559	0.0000
			Latent vs. Control	−0.293	0.7872

**Table 3:** Comparison of model performance using the four-gene signature under different scaling methods.

Scaling Method	AUC (95% CI)	Sensitivity (95% CI)	Specificity (95% CI)	Accuracy (95% CI)	Macro F1-Score (95% CI)
RobustScaler	0.9911 (0.983–0.997)	90.0% (85.5–93.8%)	89.47% (84.2–93.5%)	86.2% (81.0–90.5%)	86.2% (81.0–90.3%)
Z-score	0.9902 (0.981–0.996)	90.3% (85.8–94.0%)	88.9% (83.5–93.0%)	85.8% (80.5–90.0%)	85.7% (80.4–89.8%)
Quantile	0.9895 (0.980–0.996)	89.7% (85.2–93.5%)	89.5% (84.2–93.5%)	85.5% (80.2–89.8%)	85.5% (80.1–89.6%)

**Table 4:** Benchmark against contemporary TB diagnostic models.

Study	Statistical Model	Indication	Number of Genes	Sensitivity	Specificity	AUC
Berry et al., 2010 [19]	K-nearest neighbors	ATB vs. LTBI and HCs	393	61.67	93.75	N/A
		ATB vs. ODs	86	92	83	N/A
Kaforou et al., 2013 [20]	Difference of means	ATB vs. LTBI	27	95	90	0.98
		ATB vs. ODs	44	93	88	0.95
Anderson et al., 2014 [21]	Difference of sums	ATB vs. LTBI	42	96	91	0.984
		ATB vs. ODs	51	74	78	0.862
Laux da Costa et al., 2015 [4]	Random Forest	ATB vs. ODs	3	93	95	0.955
Lee et al., 2016 [23]	Naive Bayes	ATB vs. LTBI	3	97.9	98	0.979
Maertzdorf et al., 2016 [24]	Random Forest	ATB vs. LTBI and HCs	4	88	75	0.98
Sweeney et al., 2016 [22]	Difference of geometric means	ATB vs. LTBI and ODs and HCs	3	0.82	0.79	0·88
Sambarey et al., 2017 [8]	Linear discriminant analysis	ATB vs. LTBI and HCs and ODs	10	89.67	81.0	N/A
Leong et al., 2018 [26]	Rigid logistic regression	ATB vs. LTBI	24	93.07	94.5	0.9840
Bayaa et al., 2018 [27]	LASSO	ATB vs. HCs	6	90.9	87.8	0.94
		ATB vs. LTBI	6	90.9	88.5	0.93
Wang et al., 2019 [18]	Decision Tree	ATB vs. LTBI and HCs	3	82.4	92.4	0.806
Gliddon et al., 2021 [10]	Disease Risk Score Method	TB/LTBI	3	95	85	0.973
		TB/OD	3	95	85	0.938
Perumal et al., 2021 [32]	Simple arithmetic algorithms	HCs vs. ATB	2	90.48	66.67	0.9048
		HCs/LTBI vs. ATB	2	90.91	71.43	0.8615
		HCs vs. LTBI	2	91.67	23.81	0.5357
		LTBI vs. ATB	2	90.48	71.43	0.8367
Natarajan et al., 2022 [25]	N/A	ATB vs. LTBI	7	80–100	80–95	0.84–1.00
Sutherland et al., 2022 [28]	Mann–Whitney *U* tests	TB vs. ORD	3	0.87	0.94	0.88
Luo et al., 2022 [29]	Cforest	ATB vs. LTBI	8	93.39	91.18	0.978
Xie et al., 2024 [30]	LASSO/Random Forest	ATB vs. LTBI	2	--	--	0.994
		ATB vs. HCs	2	--	--	0.782
		LTBI vs. HCs	2	--	--	0.914
Ren et al., 2025 [31]	Support Vector Machine	ATB vs. LTBI	4	--	--	0.86
		ATB vs. HCs	4	--	--	0.99
This study (2025)	Voting Classifier	ATB vs. LTBI and HCs	4	90	89.47	0.9911

## Data Availability

Datasets are publicly available at: https://www.ncbi.nlm.nih.gov/geo/query/acc.cgi?acc=GSE19439 and https://www.ncbi.nlm.nih.gov/geo/query/acc.cgi?acc=GSE19444. (accessed on 20 September 2025).

## Supplementary Materials

The following supporting information can be downloaded at: https://www.mdpi.com/article/10.3390/diagnostics16050693/s1, Figure S1: Forest-style plot comparing diagnostic performance of transcriptomic signatures for tuberculosis; Table S1: Impact of ANOVA Threshold on Gene Selection and Model Performance; Table S2: Pairwise Pearson correlation coefficients (r) for the expression of each signature gene across different scaling methods; Supplementary Method-S1: Mathematical Details of the Machine Learning Pipeline.
