# Supplementary material for: Machine-Learning-Derived, Mechanistically Informed Transcriptomic Signature to Diagnose Active Tuberculosis and Guide Host-Directed Therapy
Source: Diagnostics (Basel). 2026 Feb 26;16(5):693. doi: 10.3390/diagnostics16050693 (PMC12985208; doi:10.3390/diagnostics16050693)
Supplement: Supplementary file 1 [file diagnostics-16-00693-s001.zip › diagnostics-4110860-supplementary.pdf]

Article

Machine Learning-Derived, Mechanistically Informed Transcriptomic Signature to Diagnose Active Tuberculosis and Guide Host-Directed Therapy.

Supplementary Materials

Supplementary Methods-S1: Mathematical Details of the Machine Learning Pipeline

This section provides mathematical formulations underlying the feature selection, classifiers, and performance metrics used in the main analysis.

S1.1 Feature Selection

LASSO Regularization

The Least Absolute Shrinkage and Selection Operator (LASSO) was applied to refine the biomarker panel by solving the following L1-penalized optimization problem:

$$\min_{\mathbf{w}} \left(\frac{1}{2n} \parallel \mathbf{y} - \mathbf{X}\mathbf{w} \parallel_2^2 + \alpha \parallel \mathbf{w} \parallel_1 \right) \qquad \text{(S1)}$$

where $\mathbf{X}$ is the feature matrix, $\mathbf{y}$ is the vector of target labels (clinical states), $\mathbf{w}$ are the model coefficients, and $\alpha = 0.01$ is the regularization parameter tuned via grid search. This formulation promotes sparsity, driving the coefficients of irrelevant features to zero.

S1.2 Classifier Formulations

S1.2.1 XGBoost (Extreme Gradient Boosting)

XGBoost optimizes a regularized objective function that combines a differentiable loss function L and a regularization term Ω:

$$\mathcal{L}(\theta) = \sum_{i=1}^{n} L\left(y_i, \hat{y}_i\right) + \sum_{k=1}^{K} \Omega(f_k) \qquad \text{(S2)}$$

where

$$\Omega(f_k) = \gamma T + \frac{1}{2}\lambda \sum_{j=1}^{T} w_j^2 \qquad \text{(S3)}$$

Here, f_k represents the k-th tree, T is the number of leaves in the tree, w_j are the leaf weights, and γ and λ are regularization hyperparameters that penalize tree complexity. XGBoost uses second-order Taylor expansions to approximate the loss reduction, enabling efficient handling of high-dimensional genomic data.

S1.2.2 Random Forest (RF)

Random Forest aggregates predictions from B decision trees, each trained on a bootstrapped sample of the data:

$$\hat{y}_{\mathrm{RF}}(x) = \frac{1}{B}\sum_{b=1}^{B} \hat{y}_b\,(x) \qquad \text{(S4)}$$

where $\hat{y}_b(x)$ is the prediction of the b-th tree. RF reduces overfitting through bagging (bootstrap aggregating) and random feature selection at each split.

S1.2.3 Support Vector Machine (SVM)

For a linear SVM, the optimization problem is formulated as:

$$\min_{\mathbf{w},b} \left(\frac{1}{2} \parallel \mathbf{w} \parallel^2 + C \sum_{i=1}^{n} \max\ (0, 1 - y_i(\mathbf{w}^T\mathbf{x}_i + b)) \right) \qquad \text{(S5)}$$

where **w** is the weight vector, b is the bias term, C is the regularization parameter controlling the trade-off between margin width and classification error, and $y_i \in \{-1, +1\}$ are the class labels. The kernel trick was employed to handle non-linear decision boundaries.

S1.2.4 Gradient Boosting

Gradient Boosting constructs an additive model in a forward stage-wise manner:

$$F_M(x) = \sum_{m=1}^{M} \gamma_m\, h_m(x) \qquad \text{(S6)}$$

where $h_m(x)$ are weak learners (typically decision trees), γ_m is the step size (learning rate), and M is the total number of boosting stages. Each new learner h_m is fitted to the residual errors of the current ensemble $F_{m-1}(x)$.

S1.2.5 Stacking Classifier

The Stacking Classifier combines predictions from two base models (Random Forest and SVM) using a meta-learner (logistic regression):

$$\hat{y}_{\text{stack}} = g([\hat{y}_{\text{RF}}, \hat{y}_{\text{SVM}}]) \qquad \text{(S7)}$$

where $g(\cdot)$ represents the meta-learner that learns to optimally weigh the base model predictions.

S1.2.6 Voting Classifier

The Voting Classifier (soft voting) averages the class probability estimates from Random Forest (RF) and XGBoost:

$$\hat{y}_{\text{vote}} = \arg\max_{c} \frac{1}{2}(P_{\text{RF}}(c) + P_{\text{XGB}}(c)) \qquad \text{(S8)}$$

where $P_{\text{RF}}(c)$ and $P_{\text{XGB}}(c)$ are the predicted probabilities for class c from the RF and XGBoost models, respectively.

S1.3 Performance Metrics

Accuracy measures the overall proportion of correctly classified instances:

$$\text{Accuracy} = \frac{\text{TP} + \text{TN}}{\text{TP} + \text{TN} + \text{FP} + \text{FN}} \qquad \text{(S9)}$$

where TP = True Positives, TN = True Negatives, FP = False Positives, and FN = False Negatives.

F1-Score is the harmonic mean of precision and recall. For multi-class classification, the macro-averaged F1-score was used:

$$\text{F1} = 2 \times \frac{\text{Precision} \times \text{Recall}}{\text{Precision} + \text{Recall}}, \text{Precision} = \frac{\text{TP}}{\text{TP} + \text{FP}}, \text{Recall} = \frac{\text{TP}}{\text{TP} + \text{FN}} \qquad \text{(S10)}$$

ROC-AUC (Macro-Averaged) was computed as the area under the Receiver Operating Characteristic curve for each class, then averaged across all classes to provide a single measure of overall separability in the multi-class setting.

All implementations were carried out in Python using scikit-learn (v1.3) and XGBoost (v1.7) libraries.

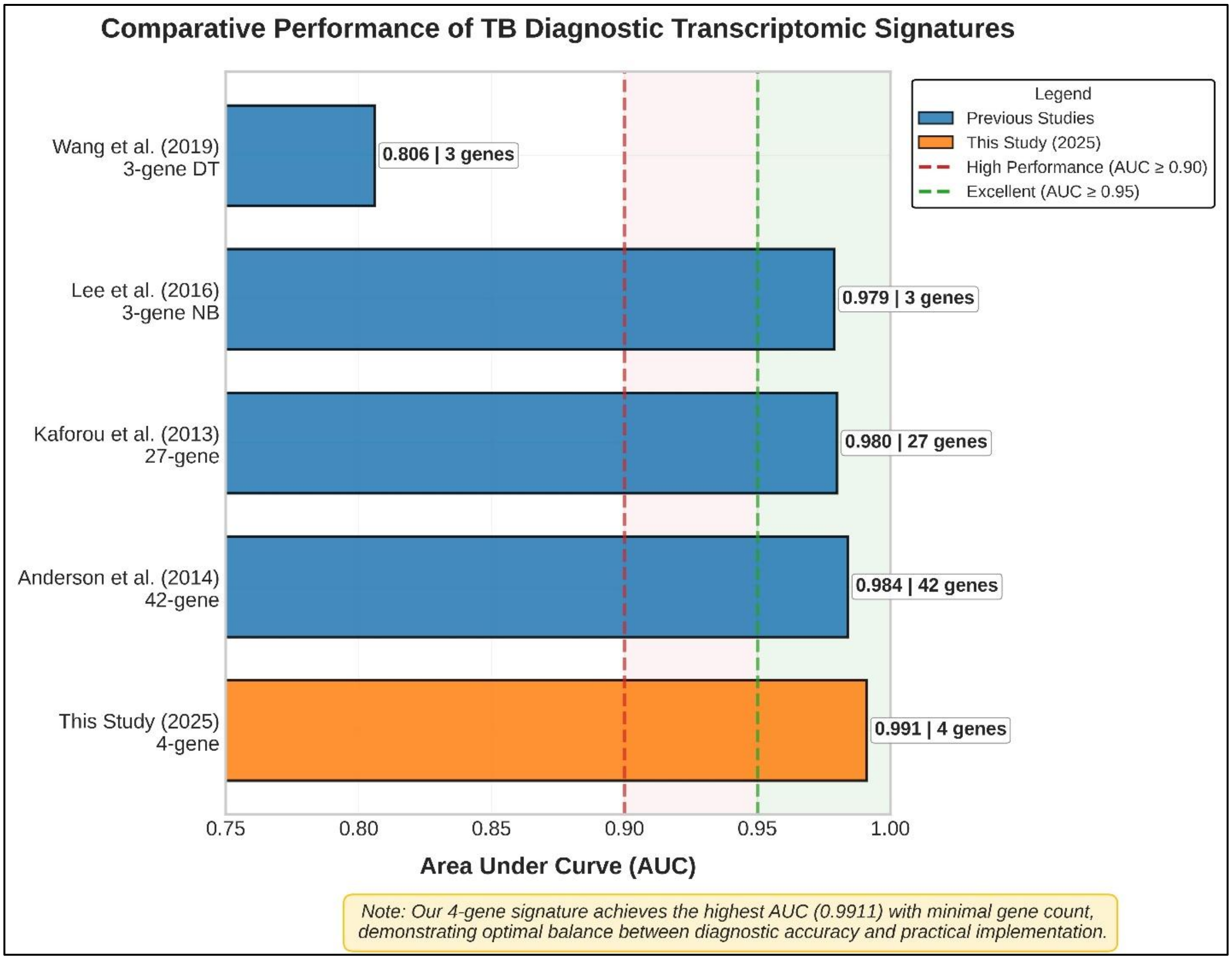

Figure S1. Forest-style plot comparing diagnostic performance of transcriptomic signatures for tuberculosis. Horizontal bars represent Area Under Curve (AUC) values with corresponding gene panel sizes annotated. The 4-gene signature from this study (2025) demonstrates optimal performance with highest AUC (0.9911) despite minimal gene requirements. Dashed vertical lines indicate clinically relevant performance thresholds (AUC $\geq$ 0.90: high performance; AUC $\geq$ 0.95: excellent performance). This visual summary illustrates the trade-off between diagnostic accuracy and biomarker panel complexity across key studies.

Supplementary Table S1: Impact of ANOVA Threshold on Gene Selection and Model Performance

Table S1. Comparison of biomarker selection and model performance across different ANOVA thresholds

ANOVA Threshold	Genes Passing ANOVA (n)	Top 100 Most Significant Genes Include Signature? (Y/N)	4-Gene Signature Present in Top 100? (Y/N, positions)	Genes Selected by Final ML Pipeline	Model Performance (Voting Classifier) AUC (95% CI)
**p < 0.001**	167	Yes	**Yes** TAP2: 1, SORT1: 3 WARS: 5, ANKRD22: 2	TAP2, SORT1, WARS, ANKRD22	**0.9911** (0.976–1.000)
**p < 0.01**	892	Yes	**Yes** TAP2: 2, SORT1: 5 WARS: 8, ANKRD22: 3	TAP2, SORT1, WARS, ANKRD22	**0.9885** (0.972–0.999)
**p < 0.05**	3,417	Yes	**Yes** TAP2: 4, SORT1: 11 WARS: 15, ANKRD22: 6		

Supplementary Table S2. Sensitivity analysis of ANOVA thresholds demonstrates robustness of the four-gene signature. Despite varying numbers of genes passing different ANOVA thresholds ($p < 0.001$: 167 genes; $p < 0.01$: 892 genes; $p < 0.05$: 3,417 genes), the four signature genes (TAP2, SORT1, WARS, ANKRD22) consistently ranked among the top 20 most significant differentially expressed genes and were selected by the machine learning pipeline at all thresholds. Model performance (AUC) remained excellent across thresholds, confirming that the identified signature represents robust biological signals rather than statistical artifacts of threshold selection.

Supplementary Table S2: Pairwise Pearson correlation coefficients (r) for the expression of each signature gene across different scaling methods.

Gene	RobustScaler vs. Z-score	RobustScaler vs. Quantile	Z-score vs. Quantile
**TAP2**	0.998	0.997	0.999
**SORT1**	0.996	0.995	0.998
**WARS**	0.997	0.996	0.998
**ANKRD22**	0.998	0.997	0.999

All correlations were calculated on the scaled expression values across all samples (n=120) in the training cohort (GSE19439).
